# The Role of Entropy in the Development of Economics

**DOI:** 10.3390/e22040452

**Published:** 2020-04-16

**Authors:** Aleksander Jakimowicz

**Affiliations:** Department of World Economy, Institute of Economics, Polish Academy of Sciences, Palace of Culture and Science, 1 Defilad Sq., 00-901 Warsaw, Poland; ajakimowicz@inepan.waw.pl

**Keywords:** entropy economics, non-extensive cross-entropy econometrics, non-ergodic ill-behaved inverse problems, general system theory, econophysics, non-linear dynamics, complex adaptive systems, *homo oeconomicus*, edge of chaos, complexity economics

## Abstract

The aim of this paper is to examine the role of thermodynamics, and in particular, entropy, for the development of economics within the last 150 years. The use of entropy has not only led to a significant increase in economic knowledge, but also to the emergence of such scientific disciplines as econophysics, complexity economics and quantum economics. Nowadays, an interesting phenomenon can be observed; namely, that rapid progress in economics is being made outside the mainstream. The first significant achievement was the emergence of entropy economics in the early 1970s, which introduced the second law of thermodynamics to considerations regarding production processes. In this way, not only was ecological economics born but also an entropy-based econometric approach developed. This paper shows that non-extensive cross-entropy econometrics is a valuable complement to traditional econometrics as it explains phenomena based on power-law probability distribution and enables econometric model estimation for non-ergodic ill-behaved (troublesome) inverse problems. Furthermore, the entropy economics has accelerated the emergence of modern econophysics and complexity economics. These new directions of research have led to many interesting discoveries that usually contradict the claims of conventional economics. Econophysics has questioned the efficient market hypothesis, while complexity economics has shown that markets and economies function best near the edge of chaos. Quantum economics has already appeared on the horizon, which recognizes money as a fundamental measurement device in the economy. The development of these sciences may indicate the need to reformulate all mainstream economics from its foundations.

## 1. Introduction

Since its emergence, economics has been strongly methodologically linked to physics. These links made neoclassical economics possible. The aim of this paper is to review these relationships over the last 150 years and to point out a number of key discoveries that have resulted from the application of methods of physics in economics and that still remain outside mainstream economics. The probable reason that they are constantly ignored is that they undermine traditional economic knowledge.

The emergence of neoclassical economics can be dated to the first half of the nineteenth century. Its birth was influenced by economic issues related to various technical projects undertaken by French engineers who employed mathematical methods in order to solve these problems. The group of engineers included Jules Dupuit (1804–1866) and Charles Minard (1781–1870), but the achievements of the former are most significant [1,2]. The economic knowledge gained in this way was supplemented in the 1870s by Leon Walras, William Stanley Jevons and Carl Menger, but it was Alfred Marshall who gave it a coherent form [3]. In an effort to raise the scientific status of economics, neo-classicists decided to transfer to it the ideas and the mathematical apparatus from the leading science of that time—energy physics—which became the nucleus of later thermodynamics. The basic concepts of physics of the mid-19th century were translated into economic language by Irving Fisher in 1892: material points (particles) became economic entities (individuals), force was replaced by marginal utility, and energy became equivalent to utility [4]. After this, the law of equilibrium could be transferred from physics to economics. In physics, an equilibrium point is determined by the maximum of the function of net energy, while the position of equilibrium in economics is determined by the maximum of the function of gain. As a result, neoclassical economics, which is still taught today, was created, but its methodological basis has been forgotten. This basis referred to thinking about markets and economies as closed systems striving to achieve a state of equilibrium.

There is no doubt that thermodynamics significantly contributed to the emergence of neoclassical economics and that in the early twentieth century it was a huge advance in science. Since then, however, economics and physics have gradually started to move away from each other. The emergence of the global financial crisis has made it clear that today we again need an economics based on physical methods, but this can no longer be 19th-century physics—it has to be replaced by 21st-century physics. This is proven by the results obtained by economics and econometrics of entropy, econophysics, complexity economics and, more recently, by quantum economics. Upgrading economic knowledge is not expensive; it is enough to abandon old, untrue dogmas.

The main aim of the paper is to examine the broadly understood impact of thermodynamics and entropy on the development of economics, which indicates the need to include in the research various types of entropy. The starting point for the analysis is entropy as a physical phenomenon, described by Rudolf Clausius, reflected in the second law of thermodynamics, which initiated the emergence of ecological economics. Next, various modifications of the entropy concept are taken into consideration. In recent decades, the science of entropy has been developing very fast. This has brought benefits to economics, in which the use of such forms of entropy as Shannon informational entropy or non-extensive Tsallis entropy has become a factor of progress. The analogies and metaphors combining entropy investigated in natural sciences with similar phenomena occurring in economic systems are also significant for the development of economic sciences [5]. However, despite the great importance of such analogies and metaphors, since the paper focuses mainly on similarities following the isomorphism principle, attempts have been made to limit the transfer of entropy formulae from physics to economics to logical homologies.

## 2. A Brief History of the Emergence and Development of the Entropy Concept

The emergence of entropy in physics was caused by an observation that in steam engines a large part of energy was lost due to friction and dissipation and, therefore, could not be converted into useful work. The research on this missing energy was conducted by Rudolf Clausius, who used the term entropy to describe it. In 1854, he presented the first mathematical definition of entropy in the following form [6] (p. 126):

(1) $$S=\frac{1}{T}Q$$

or

(2) $$\Delta S=\left(\frac{1}{T_{2}}-\frac{1}{T_{1}}\right)Q,$$

where ΔS stands for changes in entropy, while *Q* is the quantity of heat passing from the body with temperature $T_{1}$ to another body with temperature $T_{2}$. Clausius is also known for his concise and beautiful presentation of the first and the second law of thermodynamics [6] (p. 365):

1. The energy of the universe is constant.
2. The entropy of the universe tends to a maximum.

According to his other statement, the second law of thermodynamics takes the following form: *heat can never pass from a colder to a warmer body without some other change, connected therewith, occurring at the same time* [6] (p. 117).

A slightly different definition of entropy, being a measure of the molecular disorder of the system, was formulated by Boltzmann. It has the following form [7] (p. 137):

(3) $$S=k_{B}\ln W,$$

where $k_{B}$ is the Boltzmann constant, while W is the total number of microscopic states, corresponding to the macroscopic state of the system. Boltzmann entropy provides the basis for statistical mechanics, but the concept of probability, which is crucial in statistical theory, does not result directly from it. Thermodynamic probability W in formula (3) is not an ordinary probability, but an integer. However, it is possible to modify Boltzmann entropy to enable the introduction of the notion of probability distribution in Boltzmann statistics [8]. If an isolated system, consisting of N molecules belonging to *n* energy states is examined, then – assuming a fixed number of molecules and fixed values of the total energy – the total number of microscopic states of the system is given by the formula:

(4) $$W=\frac{N!}{\prod_{i=1}^{n}N_{i}!}\;.$$

Substituting (4) to (3), Boltzmann entropy is obtained in the following form:

(5) $$S=k_{B}\ln\frac{N!}{\prod_{i=1}^{n}N_{i}!}\approx -k_{B}N\overset{n}{\underset{i=1}{\sum }}p_{i}\ln p_{i}\;,$$

where pi=NiN and for a large N means the probability that the molecule is in the *i*-th energy state. Therefore, we obtain the entropy equation for the system consisting of N molecules distributed with probability distribution $p=\left(p_{1},\;p_{2},\;\ldots ,\;p_{n}\right)$ among *n* energy states.

In the mid-20th century, the concept of entropy found its application in information theory. As it turned out, thermodynamic entropy is very similar to information entropy. While the former concerns energy loss, the latter concerns data loss in information transmission systems. In 1948, Claude E. Shannon published his groundbreaking work, *A Mathematical Theory of Communication*, in which he addressed the issues of measures of information, choice, and uncertainty. In this work, he formulated the following function [9]:

(6) $$H=-K\overset{n}{\underset{i=1}{\sum }}p_{i}\log p_{i}\;,$$

where H stands for the Shannon entropy, whereas K is a positive constant, the role of which comes down only to a choice of a unit of measure. As can be easily observed, the H function represents entropy known in statistical mechanics, where $p_{i}$ denotes the probability of a system being in cell *i* of its phase space. Equation (5) therefore presents Shannon entropy, which measures uncertainty related to a probability distribution $\left(p_{1},\;p_{2},\;\ldots ,\;p_{n}\right)$. Thus, for large classical systems, Boltzmann entropy is proportional to Shannon entropy. At the same time, it should be emphasized that various types of entropy represented by Equations (1), (3), (5) and (6) are logical homologies since they present the same formal (mathematical) structure at various levels of reality. In particular, Shannon informational entropy can be treated as a more general concept compared to statistical thermodynamic entropy. In other words, this latter concept proves to be a special case of the former. In this way, the prediction of equilibrium thermodynamic properties can provide a form of statistical inference based on Shannon entropy as an information measure, while probabilities are interpreted in a subjective manner. Therefore, a reinterpretation of statistical mechanics in which statistical mechanics is based on information theory is possible. The entropy formula has a much deeper meaning than was initially believed, as it is completely independent of thermodynamics. Consequently, entropy can become a starting point for reflections, and the probability distribution maximising entropy can be used for statistical inference [10,11].

The idea of introducing the concept of entropy to information theory was put forward by John von Neumann. Shannon wondered for a long time how to name the measure of missing information he formulated (6). During a discussion, von Neumann gave him the following advice: *You should call it entropy, for two reasons. In the first place your uncertainty function has been used in statistical mechanics under that name, so it already has a name. In the second place, and more important, no one knows what entropy really is, so in a debate you will always have the advantage* [12] (p. 180). According to another version of this anecdote, von Neumann said: *Why don’t you call it entropy? In the first place, a mathematical development very much like yours already exists in Boltzmann’s statistical mechanics, and in the second place, no one understands entropy very well, so in any discussion you will be in a position of advantage!* [13] (p. 81).

## 3. The Birth of Entropy Economics

Entropy first appeared in the economic sciences in 1971, when the American economist of Romanian descent, Nicholas Georgescu-Roegen, published his opus magnum entitled *The Entropy Law and Economic Process*. This created the foundations of a novel approach to the theory of production, which consisted of applying the second law of thermodynamics to economic considerations. This gave rise to ecological economics, also called bioeconomics. Georgescu-Roegen believes that—contrary to a widely held belief—thermodynamics did not originate from striving to explain physical phenomena based on heat transfer, but from efforts aimed at understanding phenomena based on pure economics [14]. For this reason, he called thermodynamics the physics of economic values. He regards the law of entropy expressed by the second law of thermodynamics as the most economic of all the laws of physics. Economic processes (production) turn the low entropy of the original goods and services into the high entropy of the final goods and services. It is a very convincing explanation of the fact that low entropy is responsible for the utility of a given good. Therefore, only thermodynamics can explain why goods have economic value.

The constantly dwindling resources of low entropy in the environment of man are the main reason for the scarcity of goods. Production processes are characterised by the fact that they reduce the resources of low entropy, so the principal feature of economic phenomena is their irreversibility. This leads one to the conclusion that—contrary to what conventional economics holds—economic flows do not create a circular flow of income, but rather they are one-directional. High entropy can be emitted to the environment both by natural physical processes and by economic processes. The latter are characterised by the fact that they result from human purposeful actions and they are ultimately proven right by joy and satisfaction with one’s life. The economic value originates from the value that life presents to everyone. In this way, one can explain not only why people engage themselves in production processes, but also the basic goal of their economic activity, which is the preservation of mankind.

Paul A. Samuelson, a Nobel laureate in economics, said that entropy economics has changed the perception of economic processes and the creator of its concept deserves to have his achievements recognised and propagated in scientific circles. He also called him “a scholar’s scholar, an economist’s economist” [15] (p. 125). The concepts developed by Georgescu-Roegen coincided with a well-known work entitled *The Limits to Growth: A report for the Club of Rome’s Project on the Predicament of Mankind*, published in 1972 [16], i.e., at nearly the same time as the basic work on entropy economics. This coincidence of opinions bore some fruit in the form of cooperation between Georgescu-Roegen and the team of authors of *The Limits to Growth* [17]. The Club of Rome reports refer to the entropy paradigm as a major trend in economic research. Examples include *Money and Sustainability: The Missing Link. A Report from the Club of Rome–EU Chapter to Finance Watch and the World Business Academy*, published in 2012, which suggests that taking entropy into account in economic processes is a necessary condition for the success of future monetary reforms [18].

Entropy economics contributed considerably to the development of economics by emphasising the necessity of including ecological issues in the theory of economic growth. Another, rather unnoticed and unappreciated achievement of entropy economics is the fact that it created favourable conditions for the development of such novel disciplines as econophysics and complexity economics [19]. However, its creator, Nicholas Georgescu-Roegen, was never awarded the Sveriges Riksbank Prize in Economic Sciences in Memory of Alfred Nobel, despite the efforts of his followers [20]. As it turned out later, this was caused mainly by the fact that he operated outside the neoclassical paradigm, which is linked strongly with monetary reductionism, and it assumes that accelerated consumption of natural resources does not pose a considerable threat to economic growth [21]. Monetary reductionism is a frequently criticised feature of neoclassical economics. Its main idea is to reduce multidimensional relations between the natural environment and production processes to one-dimensional monetary issues. It consists in reducing non-monetary phenomena, such as health, socio-cultural, physical or ecological processes or impacts, to their monetary equivalents [22,23,24]. In this way, such features of economic phenomena as irreversibility and complexity are disregarded [25]. However, recent years have seen great progress within entropy economics concerning the development of thermodynamic techniques of modelling economic phenomena, which complement the standard econometric methods.

## 4. Thermodynamic Entropy as a Metaphor in Organization and Management Sciences

Thermodynamic entropy can be a useful metaphor in social science as a general measure of the disorder of a system. It is usually associated with a key feature of the system, which, for certain reasons, is to some extent irretrievably lost and cannot be used for its development. The management and organizational sciences use the notion of corporate entropy, which should be understood as an irretrievable loss of productive energy that cannot be transformed into useful work for the corporation. The task of enterprise management is to coordinate and integrate human work, which saves time in the entire enterprise, so that it is not lost on economic worries, complaints, beefing or partial and thus ineffective reforms. Redirecting wasted energy to productive uses is a basic condition for converting corporate entropy into useful work [26]. On the other hand, DeMarco and Lister talk about a new law, called by them the second thermodynamic law of management, according to which entropy in an organisation always grows, just like thermodynamic entropy in the universe. In their opinion, this law should be understood as follows: *most elderly institutions are tighter and a lot less fun than sprightly young companies* [27] (p. 97). They define corporate entropy as levelness or sameness. The uniformity of attitudes, appearances, and thought processes in a corporation is perceived as entropy, since it suppresses productive energy during work. This is somewhat reminiscent of Clausius’ reasoning, initially using the term of equivalence-value (Equations 1 and 2) to describe entropy. An increase in corporate entropy implies a decrease in the potential to generate energy or to perform work. The way to fight corporate entropy is as follows: *The most successful manager is the one who shakes up the local entropy to bring in the right people and let them be themselves, even though they may deviate from the corporate norm* [27] (p. 97). Social sciences also refer to similar forms of entropy, such as cultural entropy, education and school entropy, organizational entropy, as well as leadership entropy [28,29]. This last one, for instance, means less efficient and effective work and a decrease in productivity. All of them, just like corporate entropy, are based on metaphors.

## 5. Levels of Measurement as a Prerequisite for the Application of Thermodynamic Entropy in Economics

Generally speaking, the level of measurement of a variable describes a type of information contained in numbers assigned to the examined objects or subjects within a given variable. It specifies to what extent data characteristics can be mathematically modelled [30] (p. 851). Economic applications of various forms of entropy adopt Steven’s classification system, consisting of four levels of measurement: nominal, ordinal, interval, and ratio [31,32,33]. Each of them contains various properties of numbers or symbols, such as relations and operations, which specify measurements as well as the set of permissible transformations. The type of scale is defined by the group of transformations whose performance does not change the scale form. Permissible transformations include only those that do not infringe the empirical information presented by the scale. Below is a brief description of each of the scales.

1. The nominal scale is used only to classify or categorize the value of variables. It permits any one-to-one substitution of the assigned numbers. Therefore, for nominal scales the only empirical operation is the determination of equality.
2. The ordinal scale is used to order or rank the value of variables. It can be transformed using any increasing monotonic function.
3. Using the interval scale, variables can be presented in a quantitative form and used to examine usual statistical measures. The zero point, in this case, is a matter of convention or convenience since the form of the scale does not change after adding a constant. This scale can be subjected to linear transformation. A numerical value on one scale can be transformed to the value on the other scale, by using the following equation: y=ax+b.
4. A prerequisite for the existence of the ratio scale is an indication of operations enabling the determination of the following four relations: equality, rank-order, equality of intervals, and equality of ratios. After establishing such a scale, its numerical values can be transformed only by multiplying each of the values by a constant. The existence of an absolute zero is always assumed. All types of statistical measures can be applied to ratio scales. Additionally, only those scales enable logarithmic transformations.

Since the ratio scales are most commonly found in physics, their importance in research on various types of entropy is high. On the other hand, in economics and in social sciences, all four Stevens’ scale types are used, which means that entropy can be examined based on each of them. Consequently, it seems that the isomorphism principle operates here, to some extent, regardless of the assumed level of measurement of variables. Even the use of metaphors can be valuable. In establishing logical homologies, the choice of scale type is certainly important because it clarifies the considerations. As a consequence, the development of economics induced by applications of entropy has resulted in shifting the emphasis to more frequent applications of the ratio scales in this science. This explains the emergence of such scientific disciplines as econophysics and complexity economics. Thus, the separation of these transdisciplinary research fields from mainstream economics should be treated as a sign of certain cognitive conservatism. On the other hand, it must be admitted that it is not yet fully clear whether the entire economics must be transformed into econophysics or complexity economics.

Stevens used the doctrine of operationalism to develop a system for classifying the scales of measurement. Individual classes of measurement scales are determined by empirical operations required in the measurement processes and by formal (mathematical) properties of the scales. Statistical measures, which can be in a justified way applied to empirical data, depend on the scale type used for data arrangement. In the broadest sense, the measurement consists in assigning numerals to objects or events following specific rules. The concept of operationalism is attributed to Percy W. Bridgman, an American physicist and the winner of the Nobel Prize in Physics in 1946. Bridgman believed that it was possible to redefine and at the same time to specify some unobservable entities only when we could indicate physical and mental operations enabling their measurement. As an example, he provides the concept of length. In order to determine the length of any object, the performance of certain physical operations is necessary. To define the concept of length, it is necessary to indicate operations permitting length to be measured. In other words, the concept of length is explained by providing the set of operations, based on which the length is determined. Bridgman’s conclusion is as follows [34] (p. 5)*: we mean by any concept nothing more than a set of operations; the concept is synonymous with the corresponding set of operations*. The physical concept is determined by actual physical operations, and the mental concept, such as mathematical continuity, is described by mental operations. From an economic point of view, his general comments on the operational point of view are extremely interesting [34] (pp. 31–32). Adoption of operational definitions implies a far-reaching change in all our thinking habits, in that we will not be able to use concepts that are not properly explained by operations as tools in our reasoning. Initially, operational thinking may become an unsocial virtue. Later on, it has a chance to reform both the social art of conversation, and all our social relations. This is particularly true – as Bridgman claims – of popular contemporary discussions on religious or moral topics. Thus operational thinking also includes the use of different definitions of entropy in economics. Such research is expected to result in moving the issues under consideration from mainstream economics towards econophysics and complexity economics.

The application of Stevens’ classification system in economics, and in particular the growing use of the ratio scales, leads to more frequent occurrences of operational definitions of entropy in this science. As a result of such activities, the centre of thought is shifted from mainstream economics to econophysics and complexity economics. At the same time, in natural sciences, the very concept of entropy as a measure of disorder and uncertainty is constantly changing and developing, finding its expression in the theory of deterministic chaos, the theory of stochastic processes and quantum mechanics. This often involves replacing the notion of entropy with other, similar terms, better adjusted to the context of the research. These research trends are gradually penetrating economics, which results in the need to move on to econophysics and complexity economics in the further part of the paper. This does not imply the tendency to omit various definitions of entropy in these approaches, but it results from the partial transformation of this notion into related concepts such as the edge of chaos or complexity.

## 6. Thermoeconomics and Operational Definitions of Entropy in Economics

As an effect of the operationalisation of the entropy concept in economics, resulting from the introduction of the ratio scales, various definitions of entropy can be the subject of reification and be applied to specific economic phenomena, such as monetary flow in the economy. In such a case, the starting point for the discussion is the circular flow of income, in which flows of goods and services as well as factors of production are compensated for with equivalent, but monetary flows in the opposite direction. However, this process cannot be continued indefinitely, because the economy would then be a perpetual motion machine, which violates the second law of thermodynamics. Wastes, which have to be disposed of outside the economic system, are inevitably generated. This must be compensated for by an influx of new resources from the environment. For this reason, the circular flow must be supplemented with the linear throughput of matter-energy, which has to supply the constant movement of money, goods and services, as well as factors of production. Linear throughput means the inflow to the economy of low-entropy natural capital, such as solar energy, mines, wells, fisheries, croplands and the outflow of high-entropy wastes, which are no longer economically valuable. An entropic flow is a part of production processes and causes that the matter and the energy participating in such processes to become less useful [35]. Therefore, economic entropy should be linked to the utility of goods and services and factors of production. This leads to the conclusion that entropy is an inherent feature of production processes, participates in the circular flow, and as such has a direct effect on real money supply (including the consequences of inflation). It must, therefore, be to some extent a monetary phenomenon itself.

The development of operational definitions of entropy was possible within a science referred to as thermoeconomics, which deals with the application of laws of thermodynamics to study economic phenomena. The processes of entropy generation in economic systems have been thoroughly analysed by John Bryant in his book *Entropy Man* [36]. The production of entropy in the economy is formulated in a way resembling Boltzmann entropy as follows:

(7) $$S=\ln\left(\;\frac{V}{X}\;\right),$$

where V stands for the volume of economic activity, and X represents the level of the constraints for that activity. Entropy changes in the economy per unit of time can be presented using a more detailed formula [36] (Ch. 4):

(8) $$dS=\left(\omega -\omega n+1\right)\frac{dV}{V}=\left(1+\frac{1-n}{r}\right)\frac{dV}{V}\;,$$

where dVV stands for the growth rate of volume flow, ω is the lifetime coefficient, n represents the elastic index, while r is the natural rate of return. Factor (ω−ωn+1) is referred to as the marginal entropic index. After the integration of Equation (8), entropy generation per unit of time takes the following form:

(9) $$S=\left(\omega -\omega n+1\right)\ln V+C=\left(1+\frac{1-n}{r}\right)\ln V+C\;,$$

where C is a constant of integration. Economic entropy can be measured in units referred to as nats, similar to entropy in information theory, where logarithms to the base of the mathematical constant e≈2.71828 are also used.

The above entropy formulas can be used to measure money entropy. In such a case, the rate of return is approximately the long-term average or natural level of the velocity of money circulation. When defining money entropy in the economy, one of the key potential constraints that should be also considered is the level of interest rates. The demand for money is represented by a function of liquidity preference formulated by John M. Keynes in his famous book *The General Theory of Employment, Interest and Money* [37]. According to this function, the interest rate is the price of money, i.e., the price that has to be paid to get people to part with liquidity for a specific period of time. Interest can be seen as a form of value flow constraint. Thus, the change in money entropy in the economy takes the following form [36] (Ch. 6):

(10) $$dS=\frac{dG}{G}-\frac{dI}{I}\;,$$

where dGG is the rate of change in output value flow G, and dII represents the rate of change in the Index of Money Interest. In other words, the change in money entropy is the difference between the growth rate of output value and the prevailing interest rate.

Changes in the interest rate are not the only factor influencing the output value flow and entropy change, as employment factors are also of great importance. In this case, the entropy formula takes the form [36] (Ch. 7):

(11) $$dS=\left(\omega -\omega n+1\right)\frac{dv}{v}=\left(1+\frac{1-n}{r}\right)\frac{dw}{w}\;,$$

where dvv stands for the rate of change in output volume flow per head, and dww represents the rate of change in the wage rate. Taking unemployment into consideration, the analysis can also use the Phillips curve, which is well-known in economics [38,39]. In line with this relationship, a high unemployment rate is usually accompanied by low rates of wage growth and inflation, and when the unemployment rate is low, rates of wage growth and inflation tend to increase. Taking this into account, the following formula for generating entropy in the economy can be presented [36] (Ch. 7) as:

(12) $$dS=\frac{dG}{G}-\frac{dJ}{J}\;,$$

where dGG represents the rate of change in output value attributed to the labour sector, while dJJ stands for the rate of change in lost value from unemployment. In other words, a change in entropy generated in the economy is equal to the difference between the output value attributed to labour that an economy can support and the potential output value flow loss taken out through unemployment, which the economy cannot support.

## 7. Non-Extensive Cross-Entropy Econometrics

Non-extensive cross-entropy econometrics (NCEE) is one of the most interesting trends in entropy economics as it is based on power-law probability distribution and it is a method of econometric model estimation, which is particularly useful for non-ergodic ill-behaved inverse problems [40,41]. It is based on non-extensive Tsallis entropy, which is a generalisation of the widely known Boltzmann–Gibbs entropy. Tsallis entropy, $S_{q}$, is particularly useful in studying systems with strong correlations between various microstates [42,43,44,45]. It is noted as the following formula:

(13) $$S_{q}=\frac{k}{q-1}\left(1-\overset{W}{\underset{i=1}{\sum }}\;p_{i}^{q}\right),\;\;q\in R,$$

where: *k* is a conventional positive constant, W∈N is the total number of possible (microscopic) configurations, $\left\{p_{i}\right\}$ describes a discrete set of probabilities with the condition $\overset{W}{\underset{i=1}{\sum }}p_{i}=1$, and *q* is any real number. The parameter *q* is often called the Tsallis index. It is a measure of the strength of the correlation between different microstates of a system and it can take values smaller or greater than 1. If this parameter approaches 1, then the Tsallis entropy reduces to the usual Boltzmann–Gibbs entropy $\left(S_{BG}\right)$. We can write [44,45] it as:

(14) $$p_{i}^{q}=p_{i}p_{i}^{q-1}=p_{i}e^{\left(q-1\right)\ln p_{i}}\;\sim \;p_{i}\left[1+\left(q-1\right)\ln p_{i}\right],$$

hence,

(15) $$S_{1}\equiv \underset{q\to 1}{\lim}S_{q}=k\underset{q\to 1}{\lim}\frac{1-\sum_{i=1}^{W}p_{i}\;\exp\left[\left(q-1\right)\ln p_{i}\right]}{q-1}=-k\overset{W}{\underset{i=1}{\sum }}p_{i}\ln p_{i}=S_{BG},$$

where *i* denotes the number of different microstates each of which has its own probability $p_{i}$ of occurring.

There is some terminological confusion in the physics literature concerning such properties of physical quantities as additivity and extensivity. This is of particular importance in research on the entropy measure proposed by Tsallis, where those two notions are often treated as synonyms. However, in this case, non-extensivity cannot be identified with non-additivity [46,47,48,49]. In fact, the Tsallis entropy is pseudo-additive entropy of degree-q. If we have two independent systems A and B, such as the probability distribution of the composite system A+B is expressed by the formula P(A+B)=P(A)P(B), the Tsallis entropy of the A+B system is as follows:

(16) Sq(A+B)=Sq(A)+Sq(B)+(1−q)Sq(A)Sq(B)k .

This property is referred to as pseudo-additivity. If q=1, we are dealing with an additive system.

Integration of the classic econometric model with one of Tsallis non-extensive entropy, which involves a proper choice of constraints, allows for generating new data, which reduce the entropy of a system under study, thereby allowing for more precise estimation of the model parameters. This approach allows for analysing complex systems described with heavy-tailed distributions, with long memory and characterised by scale invariance. Since rare events occurring in non-ergodic systems are associated closely with heavy tails, the use of normal distribution is limited. In this manner, fractal and multifractal objects are included in considerations, which exhibit self-similarity in different temporal and spatial scales [50,51]. Therefore, power-law probability distributions become the focus of research attention; they are useful in describing systems both with low-frequency and high-frequency events.

Generalisations of the normal distribution using the non-extensive Tsallis entropy can describe ergodic and non-ergodic socio-economic systems. In the case of isolated macroscopic systems, ergodicity means that, with time, a phase trajectory runs through all the allowed microstates. On the other hand, non-ergodic systems are characterised by multi-level correlations between system microstates, with the consequent occurrence of non-extensive entropy in them, which is not a linear function of the number of possible states. There is quite convincing evidence that the amplitude and frequency of socio-economic phenomena do not substantially diverge from many extreme events occurring in nature if they are transformed by power law distribution into the same time or space scales. A distribution arising from the aggregation of statistical data depends on the nature of the phenomena under study and it results from an effect of two attractors: the power-law attractor associated with extreme events and the Gaussian attractor, which occurs in the central part of the data series. The use of pseudo-additive Tsallis statistics in modelling allows for preserving long-range correlation and the observed time-scale invariance structure of high-frequency series in the process of transformation (aggregation) of high-frequency data to a lower frequency. The differences between an approach typical of NCEE and other econometric methods become visible when aggregated data approach the normal distribution, i.e., economic systems of relatively low complexity levels are considered. In such cases, entropy econometrics referring to Gibbs–Shannon’s classic concepts gives a result similar to NCEE, because the Tsallis complexity index (q) goes to one. Similar results can be obtained with classic econometric techniques. However, NCEE methods are more useful in the case of complex systems described by Lévy processes [52]. Therefore, the non-ergodic approach is at least non-inferior to the parameter estimation methods now used in econometrics.

Entropy econometrics is particularly useful in forecasting input-output table parameters, which are presented as an inverse problem. We have an inverse problem when we use a set of observations to establish the causes that generated the set. In other words, the effects are used to establish the causes. It is the reverse of a forward problem, whose solution starts with causes, which are then used to determine the effects. A reverse problem can be defined as follows:

(17) $$d_{obs}=F\left(p\right),$$

where $d_{obs}$ is a set of noted observations, F(p) denotes the forward map, and *p* stands for the set of model parameters. It denotes the determination of the parameters of the model *p*, which generated the set of observations $d_{obs}$. When it comes to input-output tables, they are often unbalanced, due to which a researcher faces an ill-behaved (troublesome) inverse problem. In such cases, the pseudo-additive Tsallis entropy gives a numerically more stable result than the Shannon entropy. Nevertheless, Shannon’s entropy is useful for solving many other economic problems, such as developing a new interpretation for uncertainty and risk related to economic disparity [53] and analysing the quality of institutions in the European Union countries from the perspective of their influence on the speed of resource reallocation [54] or comparing the development level of the digital economy in various countries [55].

The NCEE method also enables obtaining good results for the estimation of production function parameters with constant elasticity of substitution (CES) between production factors, as it allows for demonstrating that a non-linear function CES can have an analytically closed-form solution. So far, solitons have been the best-known examples of such non-linear functions [56]. This result was achieved at the model variance minima and varying q-Tsallis complexity index when the estimated CES function was described with the power law. However, it required the previous demonstration of a relationship between the power-law distribution and the macroeconomic aggregate structure of national accounts. Moreover, calculations have demonstrated the superiority of the NCEE method over such traditional estimation techniques as Shannon entropy, the non-linear least squares, the generalized methods of moments, and the maximum likelihood approaches [57]. Non-extensive Tsallis entropy also finds a number of applications on financial markets, which includes studies concerning the distribution of return fluctuations for the Polish stock market index WIG20 [58], the origin of multifractality in the time series [59], the exchange rate return fluctuations [60], relationship between the stock market returns and corresponding trading volumes [61], the memory effect involved in returns of companies from WIG 30 index on the Warsaw Stock Exchange [62] and the asymmetry of price returns on stock and money markets [63]. Other types of entropy have also been applied to financial markets [64].

## 8. Program for the Development of Econophysics formulated by Zygmunt Rawita-Gawroński in 1958

The foundations of modern econophysics were formulated by the Polish economist Zygmunt Rawita-Gawroński. He pointed to the need to supplement the methodology of economics with some ideas of physics, mainly with the theory of stochastic processes and the non-linear dynamics he anticipated, which he called an “aleatory momentum”. Rawita-Gawroński’s article is a collection of ideas aimed at improving economics by the large-scale introduction of physics methods. This work is still relevant and it seems that it will remain so for a very long time. Although written over 60 years ago, it reads as if it was written yesterday. Rawita-Gawroński’s program of econophysical research consists of the following elements [65]:

### 8.1. General Formulation of the Econophysics Scientific Problem

The rapid development of physics and the related progress in mastering natural forces should be used to develop economic methodologies. First of all, the need to apply the concept of entropy and selected elements of thermodynamics, statistical mechanics and quantum theory is stressed.

### 8.2. A Criterion for Applying Physics Methods in Economics

The greatest benefits may be gained by economic theories with the same approach to the phenomena they study as in natural sciences. First of all, the microcosmic approach is mentioned, which has to be cleared of any metaphysical tarnish in order for the object of research to be quantitatively acceptable. This would allow for the application of mathematics. The importance of general principles applicable to all sciences is also emphasized, which indicates thinking in terms of general system theory.

### 8.3. The Principium Rationis Sufficientis as a Mental Aspect of Causality

This principle, which stems from the fundamental criterion of *praedicatus inest subjecto*, is based on divine wisdom. It is in line with Poincaré’s views, for whom the physical phenomenon is the result of a combination of many causes, but their influences cannot be distinguished and evaluated. Causality is also the basis of statistical laws, manifested in mass phenomena.

### 8.4. Development of the Classic Concept of Equilibrium Based on the Works of Walras and Pareto

It is impossible to formulate a definition of equilibrium in economics without referring to the second law of thermodynamics. All isolated systems aim at a state of equilibrium corresponding to the minimum of free energy in relation to the total energy of the system, which is equivalent to the maximum of the entropy function. Movements leading to equilibrium can be reversible if they originate from the causal laws, or are irreversible if they are a consequence of an increase in entropy and thus the result of statistical laws.

### 8.5. Types of Equilibrium in Economics

Equilibrium should be classified according to how it is reached. This can be done by the cancellation of opposing moves, by fusion or by accumulation. The first type is the mechanical equilibrium achieved by cancellation, which occurs when all movements are homogeneous. Thermodynamic equilibrium, on the other hand, is achieved by a fusion of unobservable micro-movements, which leads to a macroscopic state associated with the maximum entropy. The third type is psychological equilibrium, whose importance in economics cannot be overestimated, based on the accumulation of past states. This is a fundamental issue in psychoanalysis and somewhat similar to inertia. This equilibrium is a kind of attitude of man towards the world he wants to know.

### 8.6. Anticipation of the Definition of Complex Adaptive Systems given by Gell-Mann Several Decades Later

In the statistical equilibrium, the principle of cognitive level difference between the phenomenon and its explanation scheme applies. The schemes are used to explain the forms of macroscopic phenomena. They are temporary because they are improved during cognitive processes.

### 8.7. Anticipating the Existence of Dissipative Structures in Economics and Physics

Statistical laws are a link between stochastic processes and life phenomena. The concept of personality has no clear bottom limit and it emerges slowly as the complexity of material structures increases. However, this explanation must not overlook the extremely important moment when particle coordination occurs. The usefulness of the ideas of evolution derived from entropy gives an important clue for further research in the sense that where organization and causality begins, statistical probabilism and determinism ends.

### 8.8. The Need to Take Open Systems into Account in Economics

Physical and economic systems are not completely isolated from the environment, so there is a possibility of fluctuations that counteract the trend towards the most probable state. This weakens the operation of statistical laws, and the significance and role of fluctuations bring the researcher closer to the problems of an individual and society and the organisation of the economy.

### 8.9. Anticipation of the Emergence of Ecological Economics (Bioeconomics) as Understood by Georgescu-Roegen and its Critical Judgment

In open systems, the phenomenon of entropy does not have to express a constant, irreversible degradation of energy in nature and reaching a state of complete homogeneity of the universe, where time and space lose all sense. A decrease in system energy quality due to entropy, which means a reduction in usable energy resources, is constantly hampered by technical progress in the use of energy sources. According to the first law of thermodynamics, with a constant amount of energy in the universe, entropy is equivalent to full energy deterioration only if we assume an unchanged level of technology.

### 8.10. Similar Limitations of Cognition in Physics and in Economics

In both sciences, there is a need to abstract from a great many causes, which affect the phenomena under study to a relatively small extent and thus can be omitted (*ceteris paribus* principle). In addition, the boundaries of scientific thought are marked by certain imperfections, antinomies or contradictions, such as Heisenberg’s uncertainty principle, the unattainability of full statistical equilibrium and the impossibility of separating adjacent systems that are expressed by the same wave function or what might be called an anti-accident.

### 8.11. Collective Facts as the Basis of Complexity Economics and Quantum Economics

Methodological constructions in economics, based on deterministic physics of the nineteenth century, do not take into account uncertainty in human behaviour. On the other hand, the physics contemporary to Rawita-Gawroński took into account stochastic probabilism, which he called the aleatory momentum. The most important research problem he posed in this paper is whether methods of physics in combination with the mathematics of aleatoric phenomena will allow capturing the whole of human activities in economics. The criterion for assessing the scientific value of econophysics is its ability to describe collective facts, which are the result of many individual actions.

### 8.12. The Role of Physics in Economics Development

Postulated by physics, stochastics is connected with the problem of economic assumptions revision. It should be modified by replacing some causal or functional relations with stochastic relations.

### 8.13. The Basic Difference between Social and Physico-Chemical Sciences

The more accurate the statistical cognition and prediction in physics, the more related the condition of the object under study is to stochastic chaos. Difficulties occur only when molecule coordination occurs and causal relationships are revealed. The main role in the description of an object in social sciences is played by an organizational element and the associated causality. The aleatory momentum, associated with stochastic chaos, is much less important, but it is the main connecting factor of these sciences.

### 8.14. Sources of Econophysics Advantage over Mainstream Economics

Different world views, traditionalisms, political orientations, environmental influences, one’s own temperament, etc., place a much greater mental burden on a theorist of economics than on a physics theorist.

The well-known Italian physicist Ettore Majorana, much earlier than Rawita-Gawroński, stressed the importance of statistical laws, especially those in thermodynamics and quantum mechanics, for the development of economics and other social sciences. However, unlike the latter, he did not formulate a more specific program of economics development supported by physical methods, i.e., econophysics [66,67]. Another difference between Majorana and Rawita-Gawroński is that the former limited himself to an analogy between physics and social sciences, while the latter thought in terms of the isomorphism principle formulated by von Bertalanffy. Nevertheless, Majorana was perfectly aware of the importance of entropy for the development of social sciences. He treated it as an additive quantity. He stressed that the development of physics emphasizes the importance of statistical laws in the whole science. He also shared Sorel’s view that determinism applies only to the phenomena classified as artificial nature, which do not occur in the presence of significant degradation of energy as provided for by the second law of thermodynamics. He pointed out that this principle must be understood as a statistical law, since entropy only describes macroscopic states, while the number of internal possibilities is so large that it is impossible to isolate and explore them all. Therefore, the number of these internal possibilities is a measure of the degree of hidden indeterminacy of the system. One can only assume that they are equally probable. Analogies with social systems are rather obvious.

Majorana perceived the role of quantum mechanics in the development of social sciences somewhat differently. It is based on statistical laws, which, in his opinion, differ significantly from the classical statistical laws, where uncertainty is the result of voluntary resignation for practical reasons from establishing the initial conditions of systems in every detail. Quantum mechanics lacks objectivity in the description of phenomena because the measurement itself changes the examined system. Moreover, this theory changes the rules of the determination for internal configurations in systems, which affects such phenomena as entropy. In classical theory, the number of internal possibilities could be infinite, whereas in quantum theory there is an essential discontinuity in the description of phenomena associated with the Planck constant. This means that the number of microstates is finite, although still huge. Therefore, in quantum physics, we have probabilistic laws, which are hidden at lower levels of reality than customary statistical laws. Rawita-Gawroński also sees this problem and claims that the introduction of quantum theory into thermodynamics removes the inability to accurately determine entropy and thus changes the definition of statistical equilibrium. It seems that both Majorana and Rawita-Gawroński present here the nucleus of the idea developed later by Gell-Mann, that deterministic chaos is a mechanism that reinforces indeterminacy inherent in quantum mechanics to macroscopic levels—occurring both in physics and in economics.

## 9. Modern Econophysics

Econophysics is a transdisciplinary science based on the observation that physical and economic objects can have a common theory. Since there are some logical homologies at its base it is an exemplification of a known isomorphism principle developed by Ludwig von Bertalanffy. The emergence of transdisciplinary areas of knowledge is consistent with the general system theory paradigm which he formulated [68]. According to the isomorphism principle, there are structural similarities between objects described by different science branches. Isomorphism is not associated with analogies, which are only superficial similarities of phenomena and processes, but with logical homologies. They occur when factors affecting certain phenomena or processes are different, whereas the formal laws that govern the dynamics of apparently different objects are identical. Discovering homologies facilitates scientific work as it reduces the time needed to explain phenomena. An issue, which is still a challenge in one branch of science, may be formally similar to another in a different branch, which has long been explained. Imparting a relatively broad meaning to the concept of a system enables transferring models from one area to another. Philip Mirowski carried out a comprehensive analysis of the relationship between natural sciences, information theory and economics [69,70,71,72,73].

Mantegna and Stanley understand econophysics as activities of physicists who strive to solve economic problems by applying methods already tested in different branches of physics [74]. However, such a definition seems to be too one-sided because it assumes cognitive actions only by physicists. One must admit that studies of this type were initiated after 1995 only by physicists [75], but now an increasing number of economists can also apply modern methods and techniques originating in physics to describe markets and economies. It seems that the dynamic development of econophysics must be based on permanent cooperation between physicists and economists in solving economic problems, which may prove beneficial for the development of both economics and physics [76,77,78].

Development of a branch of science is usually measured by its ability to formulate new knowledge about reality. Progress in research can be identified both when the use of traditional methods has led to the discovery of new facts and when new scientific laws have been discovered using innovative methods. Econophysics is an attempt to develop economic science, which is based mainly on the transfer of research methods and techniques from physics to economics. Thus, in this case we are dealing with a second possibility of increasing the knowledge base. The methods of physics most frequently used in economics include non-linear dynamics, the theory of stochastic processes, cellular automata and quantum mechanics.

The very definition of econophysics shows a certain methodological primacy of physics over economics, but numerous, although less known, examples can be given to the contrary: that economics initiated the development of physics. The first power law was discovered in the late nineteenth century by the Italian economist Vilfredo Pareto, who studied income distribution in different societies. He observed a stable relationship, independent of time and space, which differed significantly from the Gaussian curve [79,80]. Income distributions were similar in different countries and periods. If N(y) denotes the number of people with the income not lower than *y*, then the power law will describe changes in individuals’ wealth under stable economic conditions:

(18) $$N\left(y\right)=C\;y^{-\alpha },$$

where *C* is a certain constant and α>0 is called the critical exponent that Pareto estimated to be 1.5. Such distributions were applied in physics much later, mainly owing to the work of Lévy and Mandelbrot.

It was similar to the random walk concept, which Bachelier developed and applied in the pricing of options in speculative markets as early as 1900 [81]. This idea appeared in physics only five years later in one of Einstein’s works [82]. In this context, the story of the discovery of the butterfly effect, which means the sensitive dependence on initial conditions, occurring in non-linear dynamical systems, is also very interesting. This was discovered by Poincaré in 1890, when he dealt with the restricted three-body problem [83]. However, this discovery was forgotten for a long time. This phenomenon was again encountered in the 1930s by the Swedish economist Palander when he studied certain models of duopoly and oligopoly [84,85,86]. They were also observed in the early 1950s in the course of numerical exploration of Goodwin’s non-linear business cycle model [87,88,89]. In physics, the butterfly effect was rediscovered only in 1963, when Edward Lorenz revealed the presence of the chaotic attractor, known today as the Lorenz attractor, in the non-linear model for atmospheric convection [90]. It can be seen that science is holistic, and the lack of cooperation between economists and physicists has caused considerable delays in its development. It is noteworthy that a certain cognitive balance between physics and economics can be seen in the formulation of entropy economics given by Georgescu-Roegen, which speaks in favour of this approach. Therefore, it should be accepted that there is an inherent two-way relationship between these sciences.

The most interesting achievements of econophysics apply to financial markets [91,92,93,94,95]. This is because these markets generate sufficiently large and precise datasets, which is a prerequisite for the effective application of physical methods. The flagship achievement here is the questioning of the efficient market hypothesis, as a result of the finding of endogenous self-organisation processes in major world stock exchanges [96]. Notable successes have also been achieved in modelling phase transitions, catastrophic and critical phenomena [97,98,99]. Furthermore, the sphere of interests of econophysicists embraces issues related to business cycles, factors of economic growth, income and wealth distributions, economic equilibrium, property markets, mechanisms of hyperinflation and the development of enterprises. Empirical research carried out within the framework of econophysics either complements or contradicts traditional economic knowledge. If the latter occurs, it is necessary to verify and update this knowledge constantly. The broad front of empirical research also forces changes in economic theory. Progress, in this case, is two-directional. On the one hand, the logical consistency of conventional economic models is being tested, while on the other, the possibility of developing new theories of markets and economies is being created.

Although econophysics and mainstream economics have the same goal of solving economic problems, due to significant methodological differences they are treated as two separate sciences [100]. Economists usually build their models on empirically untested premises that are treated as religious dogmas [101,102]. Thus, mainstream economics is largely a deductive science that uses axiomatic mathematical modelling. The application of economics to government policies regarding science and technology is particularly unreliable. Its usefulness is especially limited as far as the rational management of research and the conduct of thoughtful science policy are concerned [103]. Econophysics, on the other hand, is an inductive science which emphasizes empirical research and discovers relationships contained in data using mathematical tools and logic. Its essence is not to match observations to a priori models, but to discover the mechanisms of real economic systems. The success of econophysics as a science and the proper use of its achievements will only be possible if it has a noticeable impact on the course of economic phenomena. For this to happen, econophysical models must allow for accurate predictions and be accepted by economists as useful for economic policy.

## 10. Entropy, Ignorance and Complexity

As shown above, entropy and information are closely linked. It follows that entropy can be understood as a measure of ignorance [104]. Information and ignorance are opposites, but what one of them measures is also measured by the other. The entropy of a certain macrostate of the system is a measure of our ignorance concerning the actual microstate and it is equal to the number of bits of additional information necessary to describe that microstate, as long as all microstates in the macrostate are equally probable. This reasoning also applies when the system is not in a single macrostate, but can take many various macrostates, each with a different probability of occurrence. The entropy of the macrostates should then be averaged using appropriate weights in the form of their probabilities. Furthermore, entropy includes the number of bits of information necessary to determine a macrostate. To sum up, entropy means the sum of the average ignorance referring to a specific microstate within the macrostate and the ignorance of the macrostate itself. Thus, it can be claimed that—according to the second law of thermodynamics—specification of the state is equivalent to order, while ignorance corresponds to disorder.

The definition of entropy as a measure of ignorance may also take into account the operation of Maxwell’s demon, which controls a vessel filled with a gas at uniform temperature [105] (pp. 308–309). While individual gas molecules move at different speeds, the mean velocity of a large number of molecules is the same. After dividing the vessel into two parts, A and B, the demon sorts the molecules in such a way that faster ones are allowed to pass from A to B, and slower ones from B to A. As a result of the demon’s action, the temperature of part B increases and the temperature of chamber A decreases, without performing work, which is contradictory to the second law of thermodynamics. Heat flows from the cold gas to the hot one. The operation of the demon should be explained as follows. It can force the flow of heat from cold to hot bodies, but in addition to being able to measure molecular velocities, it must also collect information about these velocities. The entropy of the system consisting of cold and hot bodies can be reduced at the expense of increasing the number of records in the demon’s memory. However, this memory is finite and after it is filled in, it will be necessary to erase old records to make room for new ones. The erasing of the last copy of the information record causes at least such an increase in entropy that restores the operation of the second law of thermodynamics. Consequently, Gell-Mann proposes a new definition of entropy of the entire system, which does not violate the second law of thermodynamics, even when records exist. Algorithmic information content of records with relevant information should be added to entropy being a measure of ignorance. The algorithmic information content of a bit string is the length of the shortest computer program containing specific information. This means the exchange of ignorance for records. A decrease in entropy results in the information record to emerge, and then ignorance is reduced, but at the same time algorithmic information content of the record increases. However, erasing the record reduces the information it contains, but causes at least the same increase in ignorance related to the entire closed system.

The relationship between information and ignorance can be expressed by a mathematical formula. The ignorance measure I can be defined based on Shannon informational entropy, in which the multiplicative constant is omitted [106]:

(19) $$I=-\underset{r}{\sum }P_{r}\log P_{r}\;,$$

where $P_{r}$ stands for coarse-grained probability for the individual member r of the ensemble, while log means logarithm to the base 2 and, therefore, uncertainty is measured in bits. Based on this, a generalized measure of ignorance $I_{q}$ can be defined, taking the following form:

(20) $$I_{q}=-\left[\underset{r}{\sum }{\left(P_{r}\right)}^{q}-1\right]\;/\left(q-1\right)\;,$$

which is reduced to Equation (19) in the limit where q approaches 1. This measure can be applied to describe complex systems operating at the edge of chaos, which—as will be demonstrated—are crucial to understanding the functioning of markets and economies.

In order to solve problems related to the functioning of complex systems, such quantities as the effective complexity and total information are used [107]. The effective complexity should be understood as the length of a compact description of the regularities identified in the examined system, while total information is the sum of the effective complexity and an entropy term, which measures information necessary to describe the random aspects of the entity. Thus the total information describes both rule-based features and the probabilistic features of the perceived entity. Comparison of different sets of identified regularities of an entity permits us to show that the best set minimizes total information, which leads to minimizing the effective complexity. The effective complexity specified in such a way seems to be independent of the observer. Furthermore, there exists a relation between effective complexity and Bennett’s logical depth [108]. If the effective complexity of the binary string exceeds a certain clearly defined threshold, then this string has astronomically large depth. Otherwise, the depth is arbitrarily small.

To conclude this part, it is worth mentioning a certain paradox associated with the growth of knowledge. In recent years, our knowledge has been increasing exponentially, but ignorance has been growing at an even faster rate [109]. Therefore, an increase in knowledge is accompanied by an increase in entropy. Thus, total information about the examined system increases not only due to an increase in effective complexity, but also as a result of increased ignorance. However, Maxwell’s demon has limited memory, so it cannot indefinitely convert ignorance into records.

## 11. Complexity Economics

Although econophysics has a lot in common with complexity economics, it is not possible to equate these two sciences. Econophysics is a scientific discipline which consists of applying physical models and methods to study economic phenomena [110,111,112,113,114]. Complexity economics, in turn, is a broader discipline, since—apart from the fact that it contains almost all econophysics—it examines economic problems within complexity science. Since it is the opposite of equilibrium economics, it does not focus on order, determinacy, deduction or stasis, but as non-equilibrium economics, it emphasizes the importance of contingency, indeterminacy, sense-making and openness to change [115,116,117,118,119]. Equilibrium (neoclassical) economics is a special case of complexity economics. Additionally, complexity economics has derived many ideas and concepts from biology because it treats the economy as an evolutionary system [120,121].

Complexity economics is a response to certain shortcomings of conventional economics, such as the *homo oeconomicus* model, treating markets and economies as closed systems or relying on the assumption of linearity. The *homo oeconomicus* model defines a set of rules concerning human behaviour in economic processes, which include unbounded rationality, unbounded willpower and unbounded selfishness [122]. A closed system aiming at a state of thermodynamic equilibrium has become the main metaphor determining economic thinking throughout almost the entire 20th century. This assumption became the cornerstone of many elegant mathematical models of general equilibrium and certainly inspired the creators of the rational expectation hypothesis. Linearity, on the other hand, consists in reducing relations between phenomena to straight lines. If, in a certain system, a change of one variable at a given time by a certain multiple or fraction will change the same or another variable at a later time by the same multiple or fraction, then the system under consideration is linear. After plotting later values of a given variable as a function of previous values of the same or another variable, it turns out that the points lie on a straight line. These assumptions caused the cognitive gap between conventional economics and reality to grow steadily as a result of humanity’s economic development. In such conditions, the phenomenon of innovation or economic growth could only be explained by referring to the random exogenous shock.

In recent years, it has become apparent that dissipative structures can be identified in economic systems, i.e., those with steady states existing far from equilibrium. In such cases, we are dealing with non-extensive entropy, without the features of additivity, i.e., which is not a sum of values calculated for all subsystems. Entropy in an economic system is the sum of the production of entropy in the system itself and its exchange with the environment. The inflow of negative entropy from the environment can, therefore, compensate for the production of entropy in the system itself. After the introduction of certain disorders into the dissipative systems, it appears that they do not have to go to a state of equilibrium corresponding to the maximum entropy. In states far from equilibrium, orderly structures based on long-range correlations are created, and the occurring phenomena are characterized by long-term memory. The sensitivity of these systems to interactions of internal elements and environmental influences is related to the dissipation of energy [123,124]. Identification of dissipative structures in real systems was a significant advance in the development of the science of complexity and laid the foundations for another scientific breakthrough in identifying complex adaptive systems in nature.

Complexity economics treats markets and economies as complex adaptive systems that are by their very nature open, non-linear, consisting of interacting agents and exhibiting emergence. The sources of interaction are agents, i.e., mainly people, although they may also be other biological organisms or even computer programs. Complexity should be understood as the dynamic properties of a system that cause its behaviour to be non-periodic or structural changes to occur in it due to endogenous forces. A very important feature of these systems is emergence, which consists in their ability to create ordered collective phenomena, which can only be described at a level higher than that used to describe the components (basic rules). Complex adaptive systems collect information about their environment and their relationships with it, discover regularities in them, and use this information to build cognitive schemes that they use to operate in the real world. The effects of these activities generate a new, return flow of data used to evaluate the available set of cognitive patterns. As a result of this process, some of these schemes will survive and be improved, while others will be rejected [104].

Within complexity economics, markets and economies are understood as extremely dynamic, constantly evolving systems that resemble the brain, the Internet or the ecosystem in terms of their functionality. The greatest differences between complexity economics and conventional economics concern 10 issues: dynamics, agents, networks, emergence, evolution, technology, preferences, sources, elements and the horizon of predictability [125,126]. New ideas developed by complexity economics indicate that markets and economies tend to operate far from being balanced and that different agents should be modelled individually. They usually have incomplete information, make mistakes, but also have the ability to learn and adapt. Multilateral interactions between agents form networks that change over time. There is no difference between microeconomics and macroeconomics, as the latter is the emergent result of the interaction and behaviour of agents at the microeconomic level. The development of markets and economies is possible as a result of innovation, which is an essential factor for economic growth. Technological progress is endogenous to the economic system. The formation of individual preferences is a central issue in complexity economics, and agents do not necessarily have to be selfish. Complexity economics has its sources in biology, which is based on morphogenesis, structures, shapes, forms, capabilities, self-organization and life cycles. The source of conventional economics, on the other hand, is 19th-century physics, referring to balance and stability, which is why modern economics textbooks are mainly based on prices and quantities. In complexity economics, forecasting the dynamics of markets and economies is limited to the so-called Lapunov time, which is the inverse of the Lapunov exponent known in non-linear dynamics. The latter is a measure of the butterfly effect, so it determines whether the system is sensitive to initial conditions. The butterfly effect (deterministic chaos) occurs when this exponent is positive.

According to some critics, complexity science has difficulty in defining the very concept of complexity. Indeed, Horgan points to the existence of at least 45 different definitions of complexity, most of which refer to concepts as imprecise as entropy, randomness or information [127]. However, this is not evidence of the weakness of the concept of complexity, as this is the kind of differentiation that should be expected. The concept of complexity is only partially objective, as it depends on the level of knowledge of reality and the state of consciousness of a given agent. Therefore, the term is irreducibly subjective. Undoubtedly, however, all agents acting in certain conditions can be assigned shared initial knowledge, because what unites them is the search for order and regularity in a turbulent world. A certain amount of subjectivity in the definition of complexity must result from coarse-grained averaging, i.e., the accuracy with which a given agent (complex adaptive system) perceives reality. Therefore, it is best to adopt the definition of the effective complexity of an entity proposed by Gell-Mann. It should be understood as the length of a concise schema showing the regularity of this entity, which has been drawn up by the observer – the chosen complex adaptive system. The concept under consideration is, therefore, inherently and inextricably linked with the cognitive agent, and disregarding this relationship will always be a source of misunderstanding.

In the greatest discovery of complexity economics, it was proved that some markets and economies have the ability to tune in to the edge of chaos. A necessary, but not sufficient, condition is that they are complex adaptive systems. The edge of chaos is the distinguished state of a system situated between periodic and chaotic behaviour, where it has the maximum computing power. Around the edge of chaos, the complexity of the system is close to optimal and its adaptability is the greatest [128,129]. The edge of chaos may have one of the following forms: chaotic attractors and repellers, catastrophes of complexity, coexistence of attractors, sensitive dependence on parameters, final state sensitivity, effects of fractal basin boundaries, chaotic saddles [130].

## 12. Conclusions

There is no doubt that the emergence of entropy in economics has led to many surprising and important discoveries in the functioning of markets and national economies. However, not all of them have been fully accepted by mainstream economics. There are many reasons for this and they are quite well known. Furthermore, entropy has contributed to the emergence of econophysics, complexity economics, and quantum economics. This makes the situation in modern economics quite strange. If one compares it to a tree, the branches representing mainstream economics are developing more and more slowly, while the branches based on entropy are developing rapidly, creating new economic knowledge quite quickly. This disequilibrium may end in two ways: either mainstream economics will accept the achievements of econophysics, complexity economics and quantum economics, which will require—as Rawita-Gawroński noted more than 60 years ago—a revision of the content of economic assumptions, or economics will become the next physical science [131].

The concepts discussed here have contributed to the remarkable development of economics, but some weaknesses, particularly in their practical application, have not been overcome. The most important of these is the inability of econometrics, both traditional and non-extensive entropy-based, to determine the value of parameters accurately. This boundary of cognition results directly from non-linear dynamics, i.e., from the existence of deterministic chaos in the real world. Therefore, it is a result of the very nature of reality. Econometricians assume the correctness of a predetermined model with a number of unknown parameters and then try to forcefully match it to a non-stationary time series by the best choice of parameters. However, non-linear dynamics indicate that by matching any infinite precision model (stochastic or deterministic) to inherently finite precision data, non-uniqueness cannot be avoided [132]. The source of such problems is the sensitive dependence on parameters in non-linear dynamical systems, in which the parameter values associated with stable periodic orbits may be close to chaotic parameter values. Moreover, being unable to determine the initial conditions of the systems under study with infinite accuracy, we are exposed to the butterfly effect, whether we want it or not. Thus, econometricians mislead themselves and others by thinking that their models are helpful in understanding economic processes.

Some hopes for solving certain methodological problems are placed on a relatively new research trend called quantum economics [133,134]. It also appears that many economic issues can be treated as quantum phenomena. Logical homology, in this case, is a bridge connecting the dynamics of economic objects with the laws of motion of particles, which are of interest to quantum mechanics. The basic source of isomorphism here is Heisenberg’s uncertainty principle [135]. The more precisely we try to determine the position of some particle, the less precisely we are able to determine its momentum and vice versa. An example of a quantum phenomenon in economics can be the process of setting the price of a good or service. In quantum economics the value of a good is rather undefined, so the price reflecting it cannot be determined. Only making a transaction—by analogy to the uncertainty principle—is a measurement of value and allows for determining the price precisely. During this process, the exchange of money takes place and, therefore, money acts as a measuring device in the markets. Therefore, money is a fundamental element of economic analysis in quantum economics [136]. This concept is very promising [137,138,139,140,141,142], but time will tell if it will broaden our understanding of the regularities governing the markets and national economies. A number of arguments exist for including quantum mechanics in social scientific debates, since consciousness is probably a macroscopic quantum phenomenon [143].
